# Synergistic Application of Humic Acid and Microbial Fertilizers Improve Soil Quality, Reshape Microbial Network, and Enhance Wheat Yield in Coastal Saline–Alkali Soils

**DOI:** 10.3390/microorganisms13122716

**Published:** 2025-11-28

**Authors:** Lei Ma, Yudong Li, Yufeng Zhang, Yan Li, Jianlin Wei, Zhaohui Liu, Deshui Tan

**Affiliations:** 1 State Key Laboratory of Nutrient Use and Management, Institute of Agricultural Resources and Environment, Shandong Academy of Agricultural Sciences, Jinan 250100, China; 2 National Center of Technology Innovation for Comprehensive Utilization of Saline-Alkali Land, Dongying 257345, China

**Keywords:** humic acid, microbial fertilizer, coastal saline–alkali soil, extracellular enzyme activity, co-occurrence network, keystone species, soil restoration

## Abstract

Coastal saline–alkali soils represent one of the most challenging agroecosystems due to coupled chemical, physical, and biological constraints. Although humic acid (HA) and microbial fertilizers (MFs) are recognized as effective amendments, the mechanisms linking soil improvements to yield gains remain unclear. Here, a 2-year field experiment was conducted in the Yellow River Delta to assess the effects of HA, applied alone or in combination with *Bacillus subtilis* and *Trichoderma harzianum*, on soil salinity, nutrient availability, aggregate stability, microbial communities, and wheat yields. Results showed that HA application alone reduced soil electrical conductivity (EC) and total soluble salts (TSS), and enhanced aggregate mean weight diameter (MWD), leading to 40.94–55.64% higher yields. Co-application with MFs further amplified these improvements, lowering EC and TSS up to 77.04% and 73.83%, enhancing MWD by 122.50%, and raising yields by 75.79%. Soil enzyme activities (e.g., catalase, β-glucosidase, urease, and alkaline phosphatase) and fungal diversity were substantially enhanced, whereas bacterial diversity showed no significant change. Co-occurrence network analysis demonstrated that application of HA with MFs (particularly with *B. subtilis*) reshaped microbial networks by enriching modules linked to nutrient provisioning, aggregate stability, and enzyme activity, while suppressing modules associated with salinity tolerance. Keystone species such as *Lysobacter* and *Massilia* were significantly enriched and closely associated with soil chemical and aggregate improvements. Structural equation modeling further revealed that yield gains were mainly explained by reduced salinity and enhanced aggregate stability rather than nutrient provisioning. These findings provide mechanistic evidence that HA improves soil quality and wheat productivity in coastal saline–alkali soils through integrated chemical, physical, and biological pathways, and that these benefits are strengthened when combined with microbial fertilizers.

## 1. Introduction

Salinization represents a critical form of soil degradation, affecting more than 1 billion hectares worldwide [1]. This threat is especially acute in coastal regions, where shallow groundwater, intense evaporation, and seawater intrusion accelerate the formation of saline–alkali soils [2,3]. Elevated salt accumulation disrupts osmotic balance, induces ion toxicity, and weakens soil structural stability, thereby restricting crop root development, nutrient acquisition, and grain formation [3,4]. Beyond these physicochemical constraints, salinity reshapes soil microbial communities and suppresses extracellular enzyme activities, ultimately disrupting nutrient cycling and reducing ecosystem resilience [5,6]. Therefore, saline–alkali soils are characterized by coupled physical, chemical and biological impairments, underscoring the urgent need for sustainable strategies capable of simultaneously enhancing soil quality and restoring microbial functionality [7].

Organic amendments have gained increasing attention as effective strategies for reclaiming saline–alkali soils [8]. Among them, humic acid (HA), a structurally heterogeneous macromolecule, has demonstrated exceptional potential to improve soil nutrient content and structural stability [9,10]. HA has a high anionic charge density and reactive functional groups that can chelate soil cations and form organo–mineral complexes. These processes increase cation exchange capacity and help reduce soluble salts [11,12,13]. In addition, the carboxyl and phenolic hydroxyl groups of HA can release or absorb protons (H^+^), contributing to pH regulation [10]. Moreover, HA promotes macroaggregate formation through the binding of clay particles and organic matter, which improves porosity, lowers bulk density, and increases water-holding capacity [13,14,15]. Our previous study showed that application of 1000 kg ha^−1^ HA significantly increased enzyme activity and microbial diversity in fluvo-aquic soils [16]. However, several other studies have noted that HA may inhibit soil enzyme activities, indicating that its effects on soil microorganisms are context dependent [11,17]. Therefore, the specific effects of HA on soil microbial communities and the underlying mechanisms require further investigation.

Microbial fertilizers (MFs) serve as carriers of plant growth-promoting microorganisms (PGPMs) and important biological amendments for saline–alkali soil [6]. Inoculation with halotolerant microbial consortia has been shown to improve soil quality by regulating pH, enhancing nutrient availability, and stabilizing aggregates [6,18]. These benefits derive from microbially mediated processes, including nutrient mobilization through the release of organic acids and hydrolytic enzymes [19], aggregate formation via extracellular polysaccharide secretion [20], and stress alleviation through biostimulants and osmoprotectant synthesis [21]. Beyond these direct functions, MFs regulate co-occurrence networks and stimulate keystone species, thereby accelerating nutrient cycling, promoting organic matter mineralization, and suppressing soil-borne pathogens [6,22,23]. Field trials further demonstrate that MF application increases crop biomass, root activity, and grain yield in coastal saline–alkali soils [6,24]. Therefore, microbial fertilizers act not only as inoculants of beneficial microbes but also as biological drivers of soil functional restoration, complementing the physicochemical improvements achieved by HA.

Recent studies demonstrated that combined application of HA and MFs markedly enhanced soil fertility, enzyme activities and crop yield, outperforming single amendments [25,26]. HA may provide readily available carbon substrates and protective microenvironments that facilitate the establishment and persistence of inoculated microorganisms, whereas MFs may accelerate the decomposition and functional activation of HA substances, amplifying its effects [26,27]. However, most existing studies emphasize improvements in soil nutrient content or plant growth, and less attention has been given to changes in belowground microbial communities. In particular, the influence of HA and MF co-application on microbial co-occurrence networks and keystone taxa remains insufficiently explored, especially under field conditions.

In this study, we investigated the effects of HA and MF (*Bacillus subtilis* and *Trichoderma harzianum*) application on the physicochemical properties, enzyme activities, microbial community, and crop yield of coastal saline–alkali soils. We hypothesized that (i) application of HA alone could alleviate soil salinity, enhance nutrient availability and aggregate structure, (ii) co-application of HA and MFs would further restructure soil microbial communities, increase keystone taxa abundance, and thereby amplify soil physicochemical improvements, and (iii) crop yield improvement depends on integrated chemical–physical–biological mechanisms, whereby the synergistic application of HA and MFs not only enhances soil nutrient supply and reduces salinity but also drives microbial network reorganization that sustains soil–plant functional recovery. This study seeks to clarify the mechanisms by which HA and MFs interact in saline–alkali soils, and to provide guidance for developing sustainable management practices.

## 2. Materials and Methods

### 2.1. Site Description

A field experiment was initiated in 2023 at Niuzhuang Town, Dongying City, Shandong Province, China (118°28′10″ E, 37°21′24″ N). This region is characterized by a continental monsoon climate, with mean annual temperatures ranging from 11.3–12.4 °C, frost-free period of 196 days, and average annual precipitation of 556 mm predominantly concentrated in summer. Prior to experiment initiation (10 June 2023), the base properties of topsoil were as follows: pH 8.20, electrical conductivity (EC) 853 μs cm^−1^, total soluble salts (TSS) 3.11 g kg^−1^, soil organic matter (SOM) 13.59 g kg^−1^, available nitrogen (AN) 70.04 mg kg^−1^, available phosphorus (AP) 15.81 mg kg^−1^, and available potassium (AK) 123.33 mg kg^−1^.

### 2.2. Experimental Design and Soil Sampling

The field experiment was conducted in a randomized complete block design with three replicates. Each plot was 40 m^2^ (4 m × 10 m). Four treatments were established: (1) CK: control without humic acid or microbial fertilizer; (2) HA: humic acid (15 t ha^−1^); (3) HAB: humic acid (15 t ha^−1^) + *Bacillus subtilis* (15 kg ha^−1^); (4) HAT: humic acid (15 t ha^−1^) + *Trichoderma harzianum* (15 kg ha^−1^). The humic acid was supplied by Shandong Chuangxin Humic Acid Technology Co., Ltd. (Liaocheng, Shandong, China), containing 655.15 g kg^−1^ organic matter, 4.74 g kg^−1^ total nitrogen, 0.39 g kg^−1^ total phosphorus, and 1.41 g kg^−1^ total potassium. Microbial fertilizers were provided by Zhucheng Huikefeng Biotechnology Co., Ltd. (Weifang, Shandong, China), each with viable cell counts ≥ 1.0 × 10^10^ CFU g^−1^. The wheat cultivar *Jimai 60* was sown on 13 October 2023 and 15 October 2024. To achieve rapid improvement of coastal saline–alkali soil, HA was applied once before sowing in 2023 at a rate of 15 t ha^−1^. Microbial fertilizers were broadcast annually before sowing and incorporated into the 0–20 cm soil layer by plowing. All treatments received 270 kg N ha^−1^ (as urea) and 120 kg P_2_O_5_ ha^−1^ (as diammonium phosphate). Nitrogen was applied in equal amounts as basal and topdressing, while phosphorus was applied entirely as basal fertilizer.

Soil samples were taken during the wheat grain-filling stage on 5 May 2024 and 3 May 2025. From each plot, seven soil cores (5 cm diameter, 0–20 cm depth) were taken randomly and combined into one composite sample. The soil samples were immediately sealed in sterile polyethylene bags, stored on ice, and transported to the laboratory. After removing roots, straw, and small stones through a 2 mm sieve, each composite sample was divided into three subsamples. One portion was air-dried for chemical analyses (both 2024 and 2025). Another portion was stored at 4 °C for enzyme assays (2024 only). The final portion was frozen at −80 °C for DNA extraction (2024 only). In addition, undisturbed soil for soil aggregate classification (2024 only) was collected using a hand auger. Surface disturbance was carefully removed, and the intact cores were stored in rigid plastic containers to avoid structural damage.

### 2.3. Soil Properties and Wheat Yield Determination

Soil chemical properties were analyzed using air-dried samples following the methods described by Lu [28]. Soil pH and EC were measured in 1:5 and 1:2.5 (*w*/*v*) soil: water suspensions, respectively, using a pH and conductivity meter. TSS was determined gravimetrically method. SOM was quantified using the potassium dichromate–external heating method. AN was determined by alkali-hydrolysis diffusion, AP by 0.5 M NaHCO_3_ extraction with molybdenum blue colorimetry, and AK by 1 M ammonium acetate extraction followed by atomic absorption spectrometry. Soil aggregate stability was characterized by mean weight diameter (MWD) through wet sieving [29]. Soil catalase (CAT), urease (URE), β-glucosidase (BG), invertase (INV), and alkaline phosphatase (ALP) were quantified using commercial kits from Solarbio Science and Technology Co., Ltd., Beijing, China. CAT was determined using the hydrogen peroxide reduction method; URE was measured using the indigo colorimetry method; BG was determined by the p-nitrophenol colorimetry; INV was measured by the 3,5-dinitrosalicylic acid colorimetry; ALP was determined using the disodium phenyl phosphate colorimetric method. Wheat yield was obtained by harvesting three rows in each plot and measuring the grain weight. The recorded yield was then converted to a per-hectare value based on the harvested area.

### 2.4. High-Throughput Sequencing and Sequences Analysis Pipeline

Soil DNA was extracted from 0.5 g of fresh soil using the FastDNA Spin Kit for Soil (MP Biomedicals, Santa Ana, CA, USA). DNA concentration and purity were determined with a NanoDrop™ 2000 spectrophotometer (Thermo Scientific, Waltham, MA, USA). The V3–V4 region of bacterial 16S rRNA genes was amplified with primers 338F/806R, and the fungal ITS1 region was amplified using primers ITS1F/ITS2R. Amplicons were sequenced on the Illumina MiSeq platform (Illumina Inc., San Diego, CA, USA). Raw reads were processed using QIIME2 pipeline (version 2024.5) [30]. Low-quality bases were trimmed, paired-end reads merged, and sequences denoised using the DADA2 plugin (version 2024.5) to obtain amplicon sequence variants (ASVs). Bacterial and fungal taxonomic assignment was performed against the SILVA release 138 and UNITE release 9.0 reference databases, respectively. Sequences identified as chloroplasts were removed. The datasets were rarefied to 39,980 (bacteria) and 68,245 (fungi) reads per sample to normalize sequencing depth across samples. The raw 16S rRNA and ITS1 gene sequences data were deposited in the Sequence Read Archive (SRA) of NCBI under project accession number PRJNA1356464.

### 2.5. Co-Occurrence Network Analysis

Microbial co-occurrence network was built using the Random Matrix Theory (RMT)-based iNAP pipeline (http://mem.rcees.ac.cn:8081, accessed on 20 September 2025) [31]. ASVs with <50% occurrence or <0.01% relative abundance were excluded, resulting in 1578 ASVs (1479 bacteria and 99 fungi). Significant correlations (Spearman’s *R* > 0.91, *p* < 0.05) were used to generate adjacency matrices. Modules were defined using the greedy modularity algorithm. Topological roles of ASVs were classified as peripheral, connectors, module hubs, or network hubs based on Zi–Pi values [32]. The co-occurrence networks were visualized in Gephi (version 0.9.2).

### 2.6. Statistical Analysis

One-way ANOVA was used to test treatment effects on soil properties, crop yield, enzyme activities, microbial diversity, and module abundances; paired comparison of treatment means was achieved by Fisher’s LSD at *p* < 0.05. Prior to ANOVA, data were tested for normality and homoscedasticity, and all variables satisfied these assumptions. Composite indices were calculated using max–min normalization to scale individual variables between 0 and 1, and then averaged as follows: salinity index (normalized pH, EC, and TSS), nutrient provisioning index (normalized SOM, AN, AP, and AK), soil enzyme activity index (normalized CAT, BG, INV, URE, and ALP), and bacterial/fungal diversity indices (normalized Chao1 richness, Shannon diversity, and Pielou’s evenness) [33]. Spearman ‘s correlation analysis was carried to examine the relationship between soil variables. The above analyses were implemented using SPSS 20. Principal coordinates (PCoA) and mantel test were calculated in the R (version 4.5.1) package “vegan” (version 2.6.4). Random Forest analysis was employed to identify the main keystone species to predicted soil salinity, nutrient provisioning, aggregate stability, and enzyme activity using R package “randomForest” (version 4.7-1.2). The significance of the random forest model was assessed with 5000 permutations of the response variable using the “A3” package (version 1.0.0), and the significance of each predictor on response variable was assessed by using the “rfPermute” package (version 2.5.5). Structural equation modeling (SEM) was performed using the R package “lavaan” (version 0.6-20) to assess the direct and indirect effects of soil properties on wheat yield. The basic hypotheses of structural equation modeling were: HA and MFs were treated as exogenous variables that influence soil salinity, nutrient provisioning, and aggregate stability. These soil properties were then used as predictors of microbial keystone species, which, together with soil conditions, determined the final wheat yield response. SEM was fitted using the maximum likelihood method, and model fit was evaluated by χ^2^/df, CFI, TLI, RMSEA, and SRMR.

## 3. Results

### 3.1. Soil Physicochemical Properties and Crop Yields

Applied humic acid (HA) alone markedly improved soil physicochemical properties, while combined with microbial fertilizers (MFs) further enhanced these effects (Table 1). In both 2024 and 2025, saline–alkali stress was most severe in CK, whereas HA, HAB, and HAT reduced EC by 60.57–74.92% and TSS by 50.34–73.83%, respectively. Soil pH did not differ significantly between HA and CK but decreased markedly under HAB and HAT. Soil nutrient provisioning was substantially improved, with SOM increasing by 12.36–29.23%, and AN by 20.21–23.52%. AP was particularly increased under HAB, representing more than twofold increases over CK. AK increased under all amendments, with the maximum level observed under HAT. Soil structural stability was also strengthened, with MWD increasing by 80.83–122.50% in 2024. As a result, wheat yields increased by 40.94–55.64% with HA, 46.95–75.79% with HAB, and 50.08–70.69% with HAT across both years.

### 3.2. Soil Extracellular Enzyme Activities

**Figure 1:**
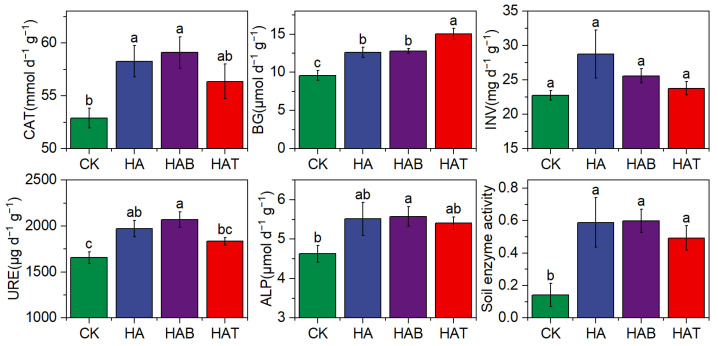
Effects of humic acid and microbial fertilizers on soil extracellular enzyme activities. CAT, Catalase; BG, β-glucosidase; INV, invertase; URE, urease; soil enzyme activity, average of standardized CAT, BG, INV, URE, and ALP. CK, control; HA, humic acid; HAB, humic acid + *Bacillus subtilis*; HAT, humic acid + *Trichoderma harzianum*. Different lowercase letters above bars indicate significant differences among treatments at *p* < 0.05 according to one-way ANOVA with Fisher’s LSD test.

Soil extracellular enzyme activities were consistently enhanced by HA and MF amendments (Figure 1). Catalase (CAT), urease (URE), and alkaline phosphatase (ALP) followed the order CK < HAT < HA < HAB, indicating the strongest stimulation under HAB. In contrast, β-glucosidase (BG) activity increased by 31.46–56.77% across all treatments, with the highest value observed in HAT. No significant differences were detected in invertase (INV) activity among treatments. When integrated into a composite index, overall soil enzyme activity was more than twice that of CK, with the greatest increases observed under HAB followed by HA.

### 3.3. Soil Microorganisms

#### 3.3.1. Community Composition and Diversity

**Figure 2:**
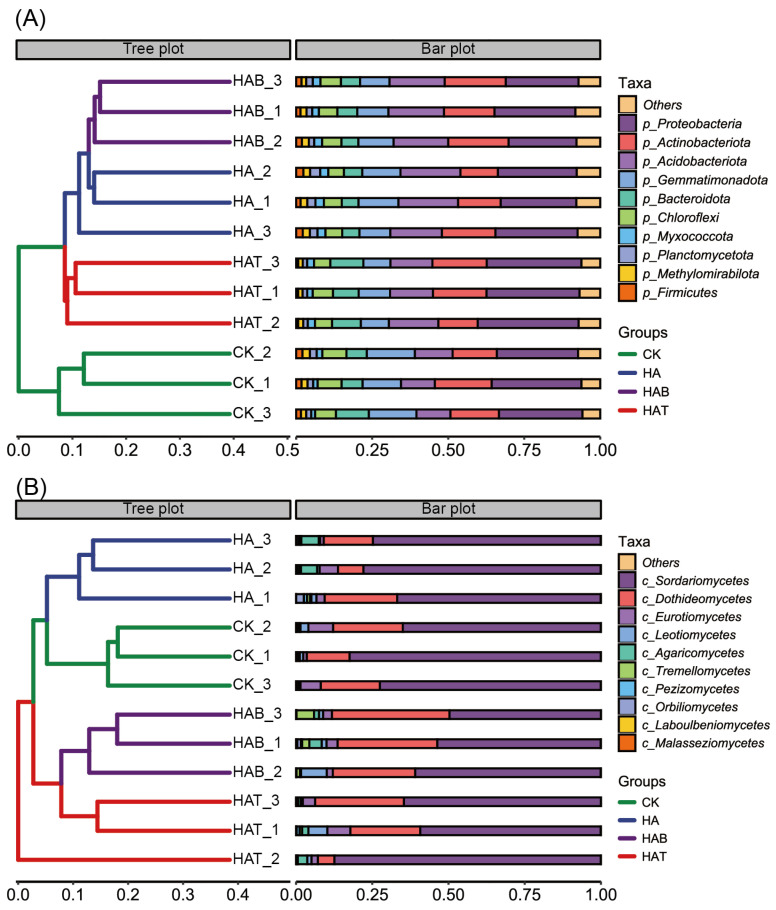
Effects of humic acid and microbial fertilizers on soil microbial community composition. (**A**) Bacterial community composition at the phylum level and hierarchical clustering of samples. (**B**) Fungal community composition at the class level and hierarchical clustering of samples. CK, control; HA, humic acid; HAB, humic acid + *Bacillus subtilis*; HAT, humic acid + *Trichoderma harzianum*. The tree plot shows Bray–Curtis-based clustering of replicates, and the bar plot represents the relative abundance of dominant taxa.

Application of HA and MFs restructured soil microbial communities markedly. At bacterial phylum level, CK were dominated by Proteobacteria (27.73%) and Actinobacteriota (16.33%), followed by Gemmatimonadota (14.62%), Acidobacteriota (11.51%), and Bacteroidota (8.10%) (Figure 2A). HA slightly decreased Proteobacteria (25.78%), but markedly increased Acidobacteriota (18.67%). By contrast, both HAB and HAT treatments induced pronounced compositional shifts. In HAB treatment, Proteobacteria (24.18%), Actinobacteriota (18.79%), and Acidobacteriota (18.06%) became co-dominant, accompanied by reductions in Gemmatimonadota (10.45%) and Bacteroidota (6.11%). HAT was characterized by a clear enrichment of Proteobacteria (31.41%) and Bacteroidota (9.53%), while Acidobacteriota declined to 14.61%. At the fungal class level, CK were dominated by Sordariomycetes (71.22%), followed by Dothideomycetes (18.20%) and Eurotiomycetes (5.17%) (Figure 2B). HA maintained a similar dominance of Sordariomycetes (68.81%), but reduced Dothideomycetes (15.16%). In HAB soils, Sordariomycetes decreased markedly to 48.33%, whereas Dothideomycetes increased to 29.30%, accompanied by enrichment of Leotiomycetes (3.19%) and Tremellomycetes (2.78%). HAT partially restored Sordariomycetes (67.72%) while increasing Eurotiomycetes (4.35%) and Leotiomycetes (2.62%).

**Figure 3:**
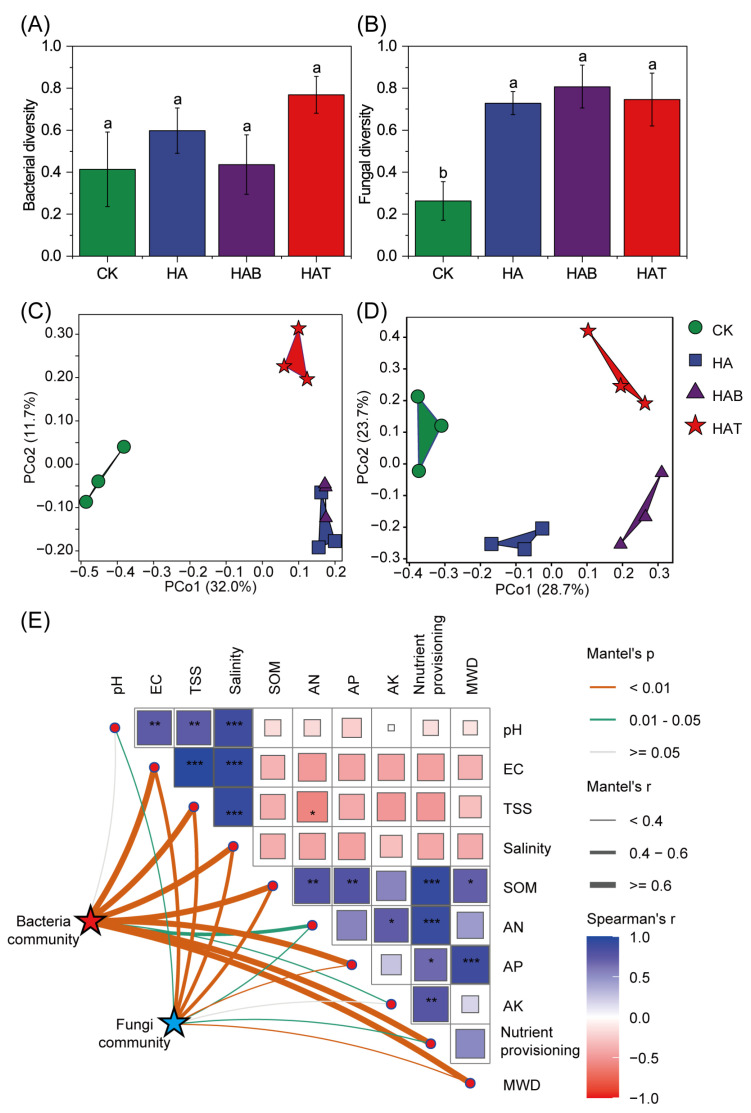
Effects of humic acid and microbial fertilizers on soil microbial diversity. (**A**) Bacterial diversity and (**B**) fungal diversity across treatments. (**C**) PCoA of bacterial communities and (**D**) PCoA of fungal communities based on Bray–Curtis distances at ASV level. (**E**) Mantel test showing correlations between microbial communities and soil physicochemical properties. CK, control; HA, humic acid; HAB, humic acid + *Bacillus subtilis*; HAT, humic acid + *Trichoderma harzianum*. Different lowercase letters above bars indicate significant differences among treatments at *p* < 0.05 (one-way ANOVA with Fisher’s LSD test). In panel (**E**), square size and color indicate Spearman’s correlation coefficients, *, **, and *** mark significant relationships at *p* < 0.05, 0.01 and 0.001, respectively. Line thickness indicates Mantel’s *r*. Orange, green, and gray edges represent significant relationships at *p* < 0.01, 0.01–0.05, and >0.05, respectively.

HA and MFs had little effects on bacterial diversity, which exhibited only minor and non-significant variation among treatments (Figure 3A). In contrast, fungal diversity increased substantially following HA and MF amendments, with HAB showing the greatest enhancement (Figure 3B). PCoA analysis showed clear shifts in community composition (Figure 3C,D). Both bacterial and fungal assemblages in amended soils separated from CK, with HA and HAB forming distinct clusters, and HAT showing a different pattern. Mantel analysis confirmed that soil microbial communities were significantly shaped by soil physicochemical properties (Figure 3E). Bacterial communities were strongly associated with salinity, aggregate stability, and nutrient provisioning, while fungal communities responded mainly to salinity.

#### 3.3.2. Microbial Network

**Figure 4:**
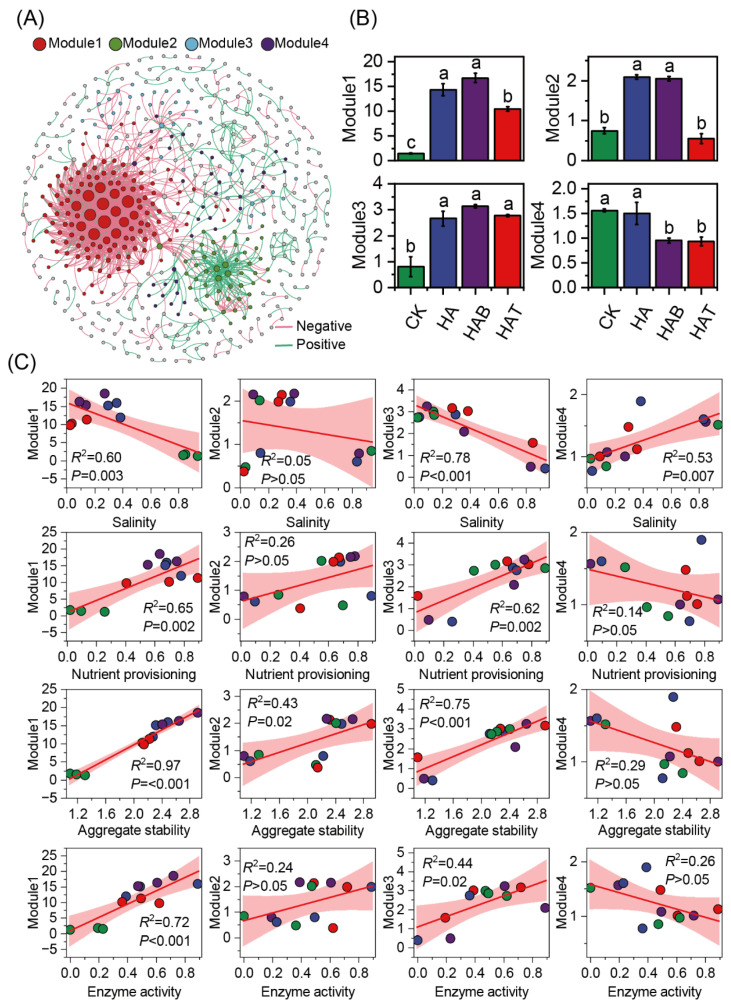
Microbial co-occurrence network modules and their relationships with soil properties. (**A**) Co-occurrence network showing four major modules (Module 1–4). Node size is proportional to degree, and edges represent significant correlations (red, negative; green, positive), grey circles indicate ASVs that do not belong to the four main modules. (**B**) Relative abundances of modules across treatments. CK, control (no amendment); HA, humic acid; HAB, humic acid + *Bacillus subtilis*; HAT, humic acid + *Trichoderma harzianum*. Different lowercase letters above bars indicate significant differences at *p* < 0.05 (one-way ANOVA with Fisher’s LSD test). (**C**) Relationships of module abundances with soil salinity, nutrient provisioning, aggregate stability, and enzyme activity.

Co-occurrence network analysis was performed to explore possible interspecific interactions between bacterial and fungal ASVs. From the network analysis, 4 major modules were identified (Figure 4A). Module 1 and module 2 were markedly enriched under HA and HAB, whereas module 3 increased across HA, HAB, and HAT (Figure 4B). By contrast, module 4 significantly declined in HAB and HAT. Regression analysis indicated that module 1 and module 3 showed strong negative associations with salinity (*R*^2^ = 0.60–0.78, *p* < 0.01) and positive associations with nutrient provisioning (*R*^2^ = 0.62–0.65, *p* < 0.01), aggregate stability (*R*^2^ = 0.75–0.97, *p* < 0.001), and enzyme activity (*R*^2^ = 0.44–0.72, *p* < 0.05) (Figure 4C). Module 2 responded weakly to soil properties, displaying only a modest positive association with aggregate stability (*R*^2^ = 0.43, *p* < 0.05). Module 4 showed limited relationships overall, with a single significant positive association with salinity (*R*^2^ = 0.53, *p* = 0.007).

**Figure 5:**
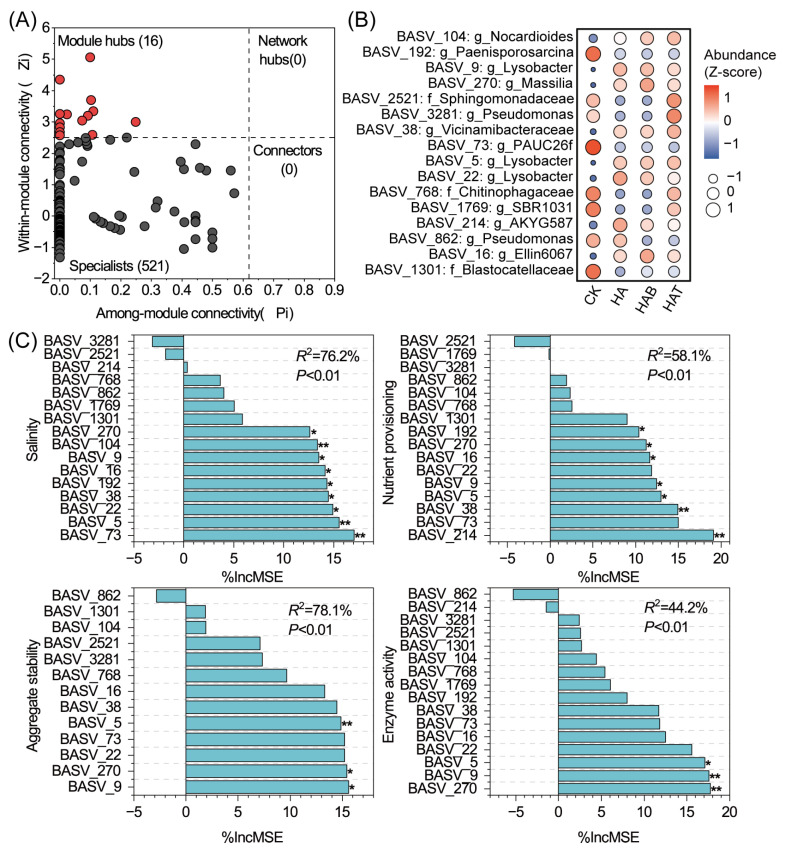
Identification of keystone taxa and their functional associations with soil properties. (**A**) Zi–Pi plot showing topological roles of ASVs within the co-occurrence network. (**B**) Heatmap of relative abundances (*Z*-scores) of keystone taxa across treatments. CK, control; HA, humic acid; HAB, humic acid + *Bacillus subtilis*; HAT, humic acid + *Trichoderma harzianum*. (**C**) Random forest analysis showing the relative importance of keystone taxa in predicting soil salinity, nutrient provisioning, aggregate stability, and enzyme activity, significance levels of each predictor are indicated by * *p* < 0.05, and ** *p* < 0.01.

Further network topology analysis identified 16 module hubs as potential keystone taxa, while no connectors or network hubs were detected (Figure 5A). These keystone taxa were mainly affiliated with *Lysobacter* (BASV_5, BASV_9, BASV_22), *Pseudomonas* (BASV_3281, BASV_862), *Paenisporosarcina* (BASV_192), *Nocardioides* (BASV_104), *Ellin6067* (BASV_16), and *PAUC26f* (BASV_73) (Figure 5B). The relative abundance of keystone taxa varied strongly among treatments. CK treatment were enriched in *Paenisporosarcina*, *PAUC26f*, Chitinophagaceae, and Blastocatellaceae but depleted in *Lysobacter*, *Massilia*, *Vicinamibacteraceae*, *AKYG587*, and *Ellin6067*. HA and HAB treatments promoted *Lysobacter*, *Massilia*, and *Ellin6067*, while reducing *SBR1031*, *Pseudomonas,* Chitinophagaceae, and Sphingomonadaceae. HAT showed a distinct enrichment of *Nocardioides*, *Pseudomonas*, *Vicinamibacteraceae*, and Sphingomonadaceae. Random forest analysis revealed that these keystone taxa strongly predicted soil functions (Figure 5C), collectively explaining 76.2% of salinity variation, 58.1% of nutrient provisioning, 78.1% of aggregate stability, and 44.2% of enzyme activity. Representative species such as *Lysobacter* (BASV_9, BASV_5), and *Massilia* (BASV_270) were particularly associated with improvements in regulation of salinity, nutrient provisioning, aggregate stability, and enzyme activity, underscoring their central ecological roles in soil functional regulation.

### 3.4. Regulatory Pathways of Crop Yields

**Figure 6:**
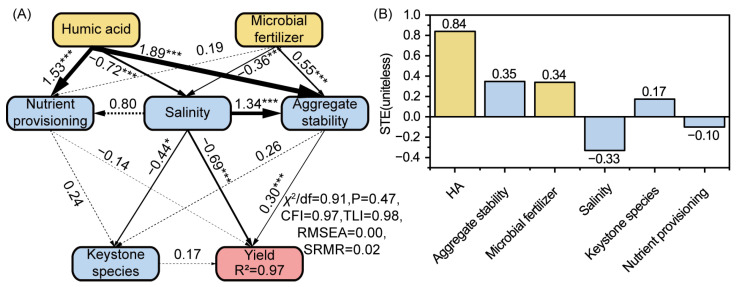
Structural equation modeling (SEM) illustrating the direct and indirect effects of humic acid, microbial fertilizers, soil properties, and keystone species on crop yields. (**A**) SEM showing pathways among treatments, soil salinity, nutrient provisioning, aggregate stability, keystone species, and yield. Numbers on arrows represent standardized path coefficients; solid lines denote significant relationships (* *p* < 0.05, *** *p* < 0.001), and dashed lines denote nonsignificant (*p* > 0.05) pathways. The model showed good fit (χ^2^/df = 0.91, *p* = 0.47, CFI = 0.97, TLI = 0.98, RMSEA = 0.00, SRMR = 0.02) and explained 97% of the variation in yield. (**B**) Standardized total effects (STE) of each factor on wheat yield.

Structural equation modeling (SEM) was used to assess the direct and indirect effects of HA and MFs on wheat yields. Results showed that HA exerted the strongest direct effects, primarily by reducing salinity (path = −0.72, *p* < 0.001), enhancing nutrient provisioning (path = 1.53, *p* < 0.001) and aggregate stability (path coefficient = 1.89, *p* < 0.001) (Figure 6A). MFs mainly improved aggregate stability (path = 0.55, *p* < 0.001) and reduced salinity (path = −0.36, *p* < 0.05). Among soil properties, salinity imposed the strongest negative effect on yield (path = −0.69, *p* < 0.001), whereas aggregate stability exerted a positive influence (path = 0.30, *p* < 0.001). Standardized total effects (STE) analysis showed that HA (STE = 0.84) was the strongest positive driver of yield, followed by aggregate stability (STE = 0.35) and MFs (STE = 0.34) (Figure 6B). In contrast, salinity (STE = −0.33) suppressed yield formation, while keystone species (STE = 0.17) and nutrient provisioning (STE = −0.10) played minor roles.

## 4. Discussion

### 4.1. Effects of Humic Acid and Microbial Fertilizers on Soil Chemical Amelioration

High salinity represents the primary constraint in saline–alkali soils, restricting crop growth through osmotic stress, ion toxicity, and nutrient immobilization [1]. Consistent with earlier findings, our results showed that HA effectively reduced soil salinity [10,34], which was also confirmed by this study. This effect is primarily attributed to the capacity of HA to complex exchangeable sodium, facilitate cation exchange, and promote the leaching of soluble salts [34,35]. Beyond its desalinating action, HA substantially improved nutrient provisioning [36]. HA inherently contains appreciable quantities of nitrogen and phosphorus that can be mineralized by soil microorganisms and subsequently absorbed by plants. Additionally, the abundant carboxyl and phenolic functional groups in HA enhance nutrient solubility and availability in the soil solution [14,37]. More importantly, co-application of HA with MFs further amplified the chemical amelioration, reducing EC and TSS by up to 77.04% and 73.83%. These findings indicate a clear synergistic effect of HA and MFs on chemical improvement of saline–alkali soils. The efficiency of HA depends on its molecular characteristics. Low–molecular-weight fractions (LMW) that contain phenolic and carboxylic groups are more reactive and biologically active than high–molecular-weight fractions (HMW) [11,38]. A recent study demonstrated that composted woody peat (main functional group is HA) with *Bacillus* enhanced dissolved organic matter transformation and improved nutrient availability [39]. Therefore, MFs may act as a biological accelerator by breaking down HA macromolecules into smaller, more active fractions, which enhances and extends the chemical modifying effects of HA.

### 4.2. Effects of Humic Acid and Microbial Fertilizers on Soil Physical Restructuring

In saline–alkali soils, electrolytes such as Na_2_CO_3_ and NaCl neutralize negative charges on soil colloids, interfere with Ca^2+^-mediated aggregation, and eventually cause aggregate breakdown [15]. In our study, HA application markedly improved soil structural stability, as reflected by a 96.67% increase in aggregate mean weight diameter (MWD). On the one hand, the abundant functional groups of HA provide additional negative charges that strengthen electrostatic repulsion between soil particles and reduce the destructive influence of Na^+^ and CO_3_^2−^ [15]. HA also strongly binds cations (e.g., Ca^2+^, Mg^2+^), promoting cation bridging, flocculation, and the re-formation of stable aggregates [11]. Furthermore, HA contains a large amount of organic binders, which can directly promote the formation of large aggregates [14]. Notably, we found that co-application of HA and *Bacillus subtilis* further enhanced aggregate stability. A recent pot experiment showed that inoculation with *Bacillus* increased macroaggregates by 65.12% and MWD by 34.47% [40]. The increase was driven by greater production of extracellular polymeric substances, which bind soil particles and strengthen aggregate stability. Therefore, HA and MFs provided both chemical binding and biological cohesion, which jointly reduced aggregate dispersion caused by soil electrolytes.

### 4.3. Effects of Humic Acid and Microbial Fertilizers on Soil Biological Reorganization

Soil salinization also leads to severe biological impairments, manifested by reduced microbial diversity and disrupted community structure [41]. In this study, bacterial diversity showed only minor changes, whereas fungal diversity increased substantially after HA application. Similarly, our previous research found that applying HA to fluvo-aquic soil can significantly increase fungal diversity, while slightly reducing bacterial diversity [16]. This difference likely reflects distinct ecological niches and substrate use strategies of bacteria and fungi. Bacteria rely mainly on easily degradable LMW, such as sugars, amino acids, and organic acids. Fungi, in contrast, have a greater enzymatic capacity to break down complex HMW organic matter (e.g., lignin, cellulose, and humified fractions) [42]. Since HA is enriched in aromatic and macromolecular structures, it provides substrates more suitable for fungi, promoting their diversification [43]. This substrate-driven partitioning also explains the contrasting regulatory patterns between bacterial and fungal community structure. Our mantel test demonstrated that bacterial community exhibited stronger associations with nutrient provisioning, salinity, and aggregate stability, reflecting their sensitivity to soil chemistry and structure changes [44,45], whereas fungal community were primarily constrained by salinity. Therefore, bacteria respond quickly to changes in soil environment, whereas fungi are more directly stimulated by complex humic substrates.

Higher salt content will also affect the interactions between microbial species and change the complexity and stability of microbial networks [5,46]. HA has been proven to increase positive cohesions and improve network stability [47]. Our co-occurrence network analysis also revealed that application of HA and MFs substantially restructured microbial interactions, leading to changes in the relative abundance of specific modules. Microbial modules are clusters of taxa with coordinated dynamics, which often reflect ecological cooperation and functional division in the community [48]. Application of HA markedly enriched modules 1 and 3, which exhibited strong positive associations with nutrient provisioning, aggregate stability, and enzyme activity, while being negatively correlated with salinity. This shift indicates that HA not only alleviated salt stress but also fostered cooperative microbial interactions that underpin soil functional recovery. By contrast, the decline of module 4 under HAB and HAT suggests that co-application of HA and MFs reduced the dominance of stress-adapted but functionally constrained taxa, thereby shifting microbial networks toward assemblages with greater functional potential.

Keystone taxa played a central role in mediating soil multifunctionality. In our study, keystone species including *Lysobacter*, *Massilia*, *Nocardioides*, *Vicinamibacteraceae*, and *Ellin6067* were consistently promoted after HA application. Numerous studies have demonstrated that *Lysobacter* serve as keystone species in the microbial network [49,50]. *Lysobacter* exhibit strong antibiotic activity, degrade chitin, and regulate soil multifunctionality by affecting microbial diversity and plant growth [50,51]. *Massilia* is an important rhizospheric microorganism, known for its strong ability to mineralize organic phosphorus and promote the growth of arbuscular mycorrhizal fungi [52,53]. *Nocardioides* [50], *Vicinamibacteraceae* [54], and *Ellin6067* [55] were also found as key species in the network, playing important roles in organic matter degradation and nutrient cycling. In contrast, the CK soils harbored keystones such as *Paenisporosarcina* and *PAUC26f*, which were less strongly linked to soil functional traits, but have been proven to be more suitable for high-salinity environments [56]. The strong correlations of keystone taxa with salinity, nutrient supply, and aggregate stability highlight their ecological importance. In particular, the enrichment of *Lysobacter* and *Massilia* after HA application was tightly coupled with improvements in structural stability and nutrient availability, suggesting that HA not only modifies soil chemistry but also selectively recruits beneficial keystone taxa that reinforce soil functionality.

### 4.4. Mechanistic Pathways of Yield Improvement

The enhanced wheat yields observed after HA and MF application can be attributed to their synergistic regulation of soil chemical, physical, and biological pathways. Structural equation modeling (SEM) identified salinity alleviation as the primary determinant of wheat yield. Similarly, results from a 3-year field experiment indicated that while organic fertilizer application was more effective in enhancing soil nutrient status, biochar exerted a stronger effect on wheat yield, primarily through reducing soil salinity and electrical conductivity [57]. This indicates that osmotic and ion stress caused by high salinity are the most serious limiting factors for wheat productivity in saline–alkali soil. Improved aggregate stability emerged as the second most influential driver, underscoring the pivotal role of soil structural integrity in supporting root proliferation, water retention, and nutrient transport under saline conditions [58]. In contrast, nutrient availability contributed more modestly and indirectly to yield enhancement, indicating that under highly degraded saline–alkali conditions, overcoming abiotic stressors takes precedence over nutrient supplementation alone. Between the two amendments, HA exerted the greater influence by simultaneously reducing salinity, enhancing nutrient accessibility, and stabilizing soil structure, thereby creating a more favorable environment for root growth and yield accumulation [25]. MFs played a complementary role, mainly by promoting aggregation and stimulating microbial activity, which enhanced the benefits of HA [25]. Notably, keystone taxa such as *Lysobacter* and *Massilia* also contributed to yield gains, but their influence appeared to operate primarily through improved salinity regulation and structural stabilization rather than direct nutrient mediation. Overall, the results point to a multi-pathway mechanism, where chemical amelioration reduces salt stress, physical restructuring strengthens soil stability, and biological processes sustain nutrient cycling and stress resilience. By combining these effects, the co-application of HA and MFs provides an effective strategy to improve saline–alkali soils and increase crop productivity.

## 5. Conclusions

Application of HA and MFs provides an effective strategy for reclaiming coastal saline–alkali soils by integrating chemical, physical, and biological improvements. HA reduced salinity and improved nutrient availability and aggregate stability. MFs further strengthened these effects by stimulating enzyme activity, enhancing fungal diversity, and reshaping soil microbial community. Network analysis revealed that co-application of HA and MFs regulated microbial modules and enriched keystone taxa such as *Lysobacter* and *Massilia*, which were closely associated with improved soil properties. Structural equation modeling confirmed that yield increases were mainly driven by reduced salinity and improved aggregate stability. In conclusion, the co-application of HA and MFs offers a promising strategy for the sustainable management of coastal saline–alkali soils by synergistically restoring soil functions and enhancing crop productivity.

## Figures and Tables

**Table 1:** Effects of humic acid and microbial fertilizers on soil physicochemical properties and wheat yield.

Year	Treatment	CK	HA	HAB	HAT
2024	pH	8.19 ± 0.04 a	8.18 ± 0.02 a	8.06 ± 0.04 b	8.02 ± 0.02 b
	EC (µs cm^−1^)	865.00 ± 16.53 a	341.00 ± 13.32 b	289.67 ± 8.95 bc	237.00 ± 27.78 c
	TSS (g kg^−1^)	2.92 ± 0.06 a	1.45 ± 0.03 b	1.39 ± 0.02 b	1.25 ± 0.02 c
	Salinity	0.87 ± 0.03 a	0.34 ± 0.03 b	0.17 ± 0.05 c	0.07 ± 0.04 c
	SOM (g kg^−1^)	13.84 ± 0.06 b	15.55 ± 0.19 a	15.54 ± 0.31 a	15.46 ± 0.58 a
	AN (mg kg^−1^)	70.67 ± 3.75 b	87.29 ± 1.52 a	83.84 ± 2.20 ab	85.77 ± 7.43 a
	AP (mg kg^−1^)	12.52 ± 0.81 c	27.15 ± 1.01 b	35.23 ± 1.74 a	24.19 ± 1.99 b
	AK (mg kg^−1^)	136.33 ± 9.06 b	179.33 ± 5.21 a	150.67 ± 3.38 b	179.00 ± 5.57 a
	Nutrient provisioning	0.13 ± 0.07 b	0.71 ± 0.03 a	0.65 ± 0.06 a	0.67 ± 0.14 a
	MWD (mm)	1.20 ± 0.06 c	2.36 ± 0.06 b	2.67 ± 0.15 a	2.17 ± 0.03 b
	Wheat yield (kg ha^−1^)	4666.50 ± 292.09 c	7262.71 ± 114.80 b	8203.37 ± 121.64 a	7965.15 ± 229.97 a
2025	pH	8.21 ± 0.03 a	8.16 ± 0.03 a	8.07 ± 0.01 b	8.07 ± 0.02 b
	EC (µs cm^−1^)	1042.33 ± 54.39 a	257.67 ± 26.36 b	239.33 ± 20.54 b	269.67 ± 34.12 b
	TSS (g kg^−1^)	3.21 ± 0.09 a	1.05 ± 0.08 b	0.84 ± 0.05 b	1.00 ± 0.04 b
	Salinity	0.91 ± 0.08 a	0.25 ± 0.03 b	0.08 ± 0.03 c	0.12 ± 0.02 bc
	SOM (g kg^−1^)	13.02 ± 0.09 b	16.77 ± 0.77 a	15.91 ± 0.28 a	15.59 ± 0.43 a
	AN (mg kg^−1^)	52.25 ± 0.05 b	65.33 ± 2.04 a	62.67 ± 1.23 a	64.75 ± 1.09 a
	AP (mg kg^−1^)	12.00 ± 0.21 c	15.23 ± 1.35 bc	25.04 ± 0.68 a	16.14 ± 1.97 b
	AK (mg kg^−1^)	148.33 ± 10.27 b	160.33 ± 8.65 ab	168.67 ± 4.06 ab	175.00 ± 6.03 a
	Nutrient provisioning	0.11 ± 0.05 b	0.59 ± 0.10 a	0.72 ± 0.04 a	0.61 ± 0.03 a
	Wheat yield (kg ha^−1^)	4283.00 ± 102.61 b	6036.33 ± 124.90 a	6293.75 ± 252.42 a	6426.67 ± 658.77 a

Values represent means ± standard errors (*n* = 3). Different lowercase letters within the same row indicate significant differences among treatments at *p* < 0.05 according to one-way ANOVA with Fisher’s LSD test. CK, control; HA, humic acid; HAB, humic acid + *Bacillus subtilis*; HAT, humic acid + *Trichoderma harzianum*. EC, electrical conductivity; TSS, total soluble salts; Salinity, average of standardized pH, EC, and TSS; SOM, soil organic matter; AN, available nitrogen; AP, available phosphorus; AK, available potassium; Nutrient provisioning, average of standardized SOM, AN, AP and AK; MWD, soil aggregate mean weight diameter.

## Data Availability

The raw sequencing reads were deposited in the NCBI Sequence Read Archive (SRA) database (Accession Number: PRJNA1356464). Further inquiries can be directed to the corresponding authors upon reasonable request.
